# The efficacy and safety of anti-PD-1/PD-L1 antibody therapy versus docetaxel for pretreated advanced NSCLC: a meta-analysis

**DOI:** 10.18632/oncotarget.23279

**Published:** 2017-12-15

**Authors:** Guanghong Huang, Xin Sun, Dapeng Liu, Yunfeng Zhang, Boxiang Zhang, Guodong Xiao, Xiang Li, Xiao Gao, Chenhao Hu, Meng Wang, Hong Ren, Sida Qin

**Affiliations:** ^1^ Department Two of Thoracic Surgery, The First Affiliated Hospital of Xi′an Jiaotong University, Xi′an, Shaanxi 710061, P.R. China

**Keywords:** NSCLC, PD-1/PD-L1, meta-analysis, Immune checkpoint inhibitor

## Abstract

Antibodies against the immune checkpoint proteins PD-1 and PD-L1 are novel therapeutic drugs for the treatment of advanced non-small cell lung cancer (NSCLC). Many clinical trials involving these drugs achieved breakthroughs in patients previously treated for advanced NSCLC. However, the results of these clinical studies are not consistent. In this report, we performed a meta-analysis to assess the efficacy and safety of anti-PD-1/PD-L1 antibodies compared with docetaxel treatment for advanced NSCLC patients from 5 randomized clinical trials. We demonstrated that the patients in anti-PD-1/PD-L1 antibody therapy groups had significantly longer overall survival (OS) (HR = 0.69, 95% CI 0.63–0.75, *P* < 0.05) and progression-free survival (PFS) (HR = 0.76, 95% CI 0.63–0.92, *P* < 0.05) than those in chemotherapy groups, especially PD-L1 positive patients. Anti-PD-1/PD-L1 antibodies improved the objective response rate (ORR) compared with docetaxel (OR = 1.64, 95% CI 1.19–2.26, *p* < 0.05). In addition, the anti-PD-1/PD-L1 antibody therapy had fewer treatment-related adverse events (AEs) (OR = 0.33, 95% CI 0.28–0.39, *P* < 0.05) than docetaxel, especially the grade ≥3 AEs (OR = 0.18, 95% CI 0.12–0.28, *P* < 0.001). In conclusion, our study revealed that, compared with docetaxel, anti-PD-1/PD-L1 antibody therapy improved clinical efficacy and safety in previously treated advanced NSCLC patients. This therapy may be a promising treatment for advanced NSCLC patients.

## INTRODUCTION

Lung cancer is one of the most common malignancies and is the leading cause of cancer-related deaths worldwide [1]. Each year, 1.8 million new cases of lung cancer are diagnosed, and 1.6 million people die as a result of this disease [1, 2]. Non-small cell lung cancer (NSCLC) accounts for approximately 85% of all lung cancers. When diagnosed, about two-thirds of NSCLC patients are at an advanced stage. Patients with advanced NSCLC have a very poor prognosis, and the mean overall survival is less than one year [3]. The primary treatment for advanced NSCLC is chemotherapy or targeted therapy. Platinum-based chemotherapy is the first-line treatment for patients with stage IIIB-IV NSCLC [4], but patients often suffer from severe adverse events and limited drug efficacy [3]. Docetaxel is one of the most commonly used second-line regimens for NSCLC. It prolongs survival of patients and relieves symptoms of the disease. However, it also causes some severe side-effects, such as neutropenia, anemia, and asthenia [4, 5]. Therefore, scientists and doctors are constantly investigating new treatments for advanced NSCLC. In the past few years, targeted therapies, such as epidermal growth factor receptor (EGFR) and anaplastic lymphoma kinase (ALK) receptor tyrosine kinase inhibitors, have achieved great success in the treatment of NSCLC. They effectively control tumor growth in patients harboring specific genetic mutations and rearrangements. Unfortunately, many patients cannot benefit from targeted therapy because they do not have the driver mutation [6]. In addition, in NSCLC patients who have undergone effective chemotherapy or targeted therapy, tumor progression may occur due to drug resistance, resulting in limited treatment options. Therefore, it is necessary to explore a new way of treating these patients in order to prolong their survival time and improve their quality of life.

Immunotherapy is emerging as a promising therapeutic strategy for the treatment of NSCLC. Cancer immunotherapy aims to restore the immune responses of CD4+ and CD8+ T cells, enabling them to function in an anti-tumor manner [7]. Immunotherapy for NSCLC involves two types of therapeutic agents: allogeneic vaccines (e.g., Liposomal BLP25, MAGE-A3, EGF, Belagenpumatucel-L, Tergenpumatucel-L, and TG4010) and immune checkpoint inhibitors (e.g., anti-CTLA-4 and anti-PD-1/PD-L1 antibodies) [7]. However, almost all phase II or phase III clinical trials involving vaccines failed to prolong the overall survival for vaccinated patients. In contrast, many clinical trials involving anti-PD-1/PD-L1 antibodies achieved breakthroughs for previously treated patients with advanced NSCLC.

Programmed death protein-1 (PD-1) receptor is expressed on activated T cells (especially on T_Reg_ cells), which is engaged by the tumor-expressed ligands PD-L1/L2 to inhibit T-cell activation and promote tumor immune escape [8]. Anti-PD-1/PD-L1 antibodies block the interaction of PD-1 with its ligand PD-L1 to activate T cells and reverse immune escape. To date, numerous clinical trials have validated the efficacy of the treatment of various malignant tumors, such as melanoma, non-small-cell lung cancer, and renal-cell carcinoma [8, 9]. The outcomes of the clinical trials for NSCLC demonstrate that these antibodies can prolong patients’ survival and improve their quality of life, thus providing a promising therapeutic strategy for NSCLC patients.

Although several phase II/III randomized clinical trials have been conducted to assess the efficacy and toxicity of anti-PD-1/PD-L1 antibodies for previously treated patients with advanced NSCLC, outcomes such as progression-free survival (PFS) seem to be controversial. Several previously published meta-analyses have analyzed the efficacy and toxicity of anti-PD-1/PD-L1 antibodies [6, 10, 11], but none of them compared anti-PD-1/PD-L1 antibodies with the second-line chemotherapy, docetaxel, for pretreated advanced NSCLC patients. In addition, the importance of PD-L1 expression should also be analyzed in the treatment of NSCLC with anti-PD-1/PD-L1 antibodies [11]. Therefore, we performed this meta-analysis systematically utilizing data from the published literature to evaluate the efficacy and safety of anti-PD-1/PD-L1 antibodies versus docetaxel in previously treated advanced NSCLC patients.

## RESULTS

### Summary of included studies

**Figure 1:**
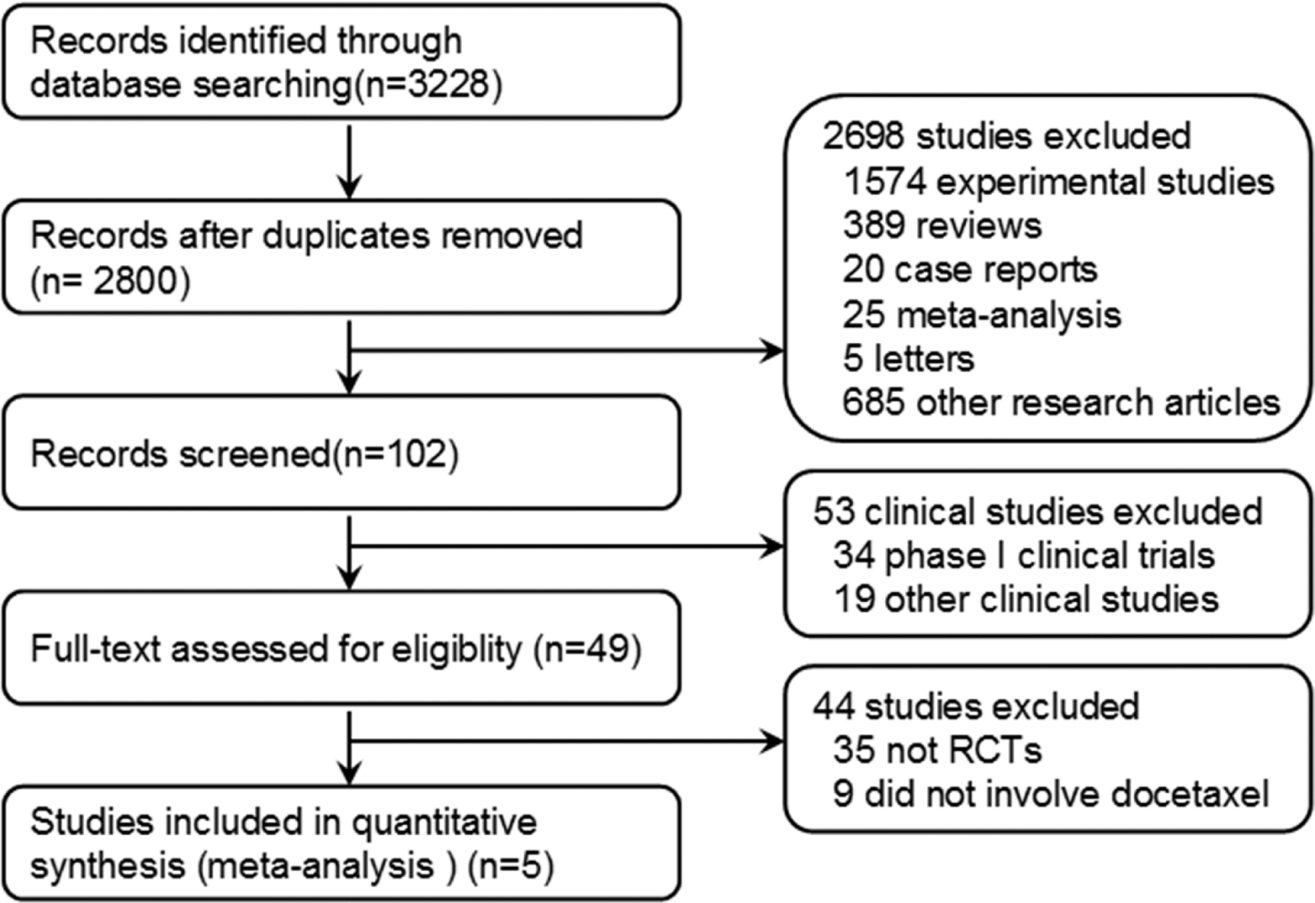
Flowchart of selecting relevant studies

Two investigators independently identified the articles eligible for further review by screening titles and abstracts. As a result, a total of 3228 records were identified according to the primary search strategy; 2800 records remained after removing the duplicates; 2698 records were removed after screening; 53 were excluded after screening the titles and abstracts; and 44 studies were excluded after reviewing each publication. Finally, we enrolled 5 published clinical trials involving a total of 3025 patients. The flow chart of our study is shown in Figure 1.

The characteristics of the 5 included studies are listed in Table 1 [12–16]. Of the 5 studies enrolled, two articles were published in 2015, and the other three were published in 2016. All the trials were randomized, controlled, open-labeled clinical trials. The POPLAR study was in phase II; the KEYNOTE-010 was in phase II/III; and the remaining 3 studies were all in phase III. The POPLAR [14] and OAK [16] studies involved the anti-PD-L1 antibody (atezolizumab) versus the second-line chemotherapy docetaxel for previously treated advanced NSCLC, while anti-PD-1 antibodies (nivolumab and pembrolizumab) were involved in the CheckMate-057 [12], CheckMate-017 [13] and KEYNOTE-010 [15] studies. Table 1 summarizes the characteristics of the included studies and agents. In addition, as the participants of the randomized clinical trial KEYNOTE-010 were assigned (1:1:1) with a central interactive voice-response system to receive pembrolizumab at 2 mg/kg or 10 mg/kg or docetaxel at 75 mg/m^2^, the KEYNOTE-010 analysis included two studies with different doses of treatment agents compared with the docetaxel group [15]. We assessed the quality of each study included in this analysis according to the Jadad score, which mainly focuses on the randomization, blinding, and follow-up.

**Table 1:** Characteristics of the 5 RCTs comparing anti-PD-1/anti-PD-L1 therapy with docetaxel

Name of RCTs	Author and Year	Registered No.	Phase of trial	Study arms	No. Of patients	Tumor histology	Jadad Score
POPLAR	Louis Fehrenbacher, 2016	NCT01903993	II	Atezolizumab 1200mg, IV q3w	144	NSCLC	3
				Docetaxel 75mg/m2, IV q3w	143		
OAK	Achim Rittmeyer, 2016	NCT02008227	III	Atezolizumab 1200mg, IV q3w	425	NSCLC	3
				Docetaxel 75mg/m2, IV q3w	425		
CheckMate 057	H. Borghaei, 2015	NCT01673867	III	Nivolumab 3 mg/kg, IV q2w	292	Non-squamous	3
				Docetaxel 75mg/m2, IV q3w	290		
CheckMate 017	Julie Brahmer, 2015	NCT01642004	III	Nivolumab 3 mg/kg, IV q2w	135	Squamous	3
				Docetaxel 75mg/m2, IV q3w	137		
KEYNOTE-010	Roy S Herbst, 2016	NCT01905657	II/III	Pembrolizumab 2mg/Kg, IV q3w	345	NSCLC	3
				Pembrolizumab 10mg/Kg, IV q3w	346		
				Docetaxel 75mg/m2, IV q3w	343		

IV = intravenous infusion, NSCLC = nonsmall-cell lung cancer, q2w = 2 weeks using a time, q3w = 3 weeks using a time.

### Anti-PD-1/PD-L1 antibodies prolonged overall survival compared with docetaxel

**Figure 2:**
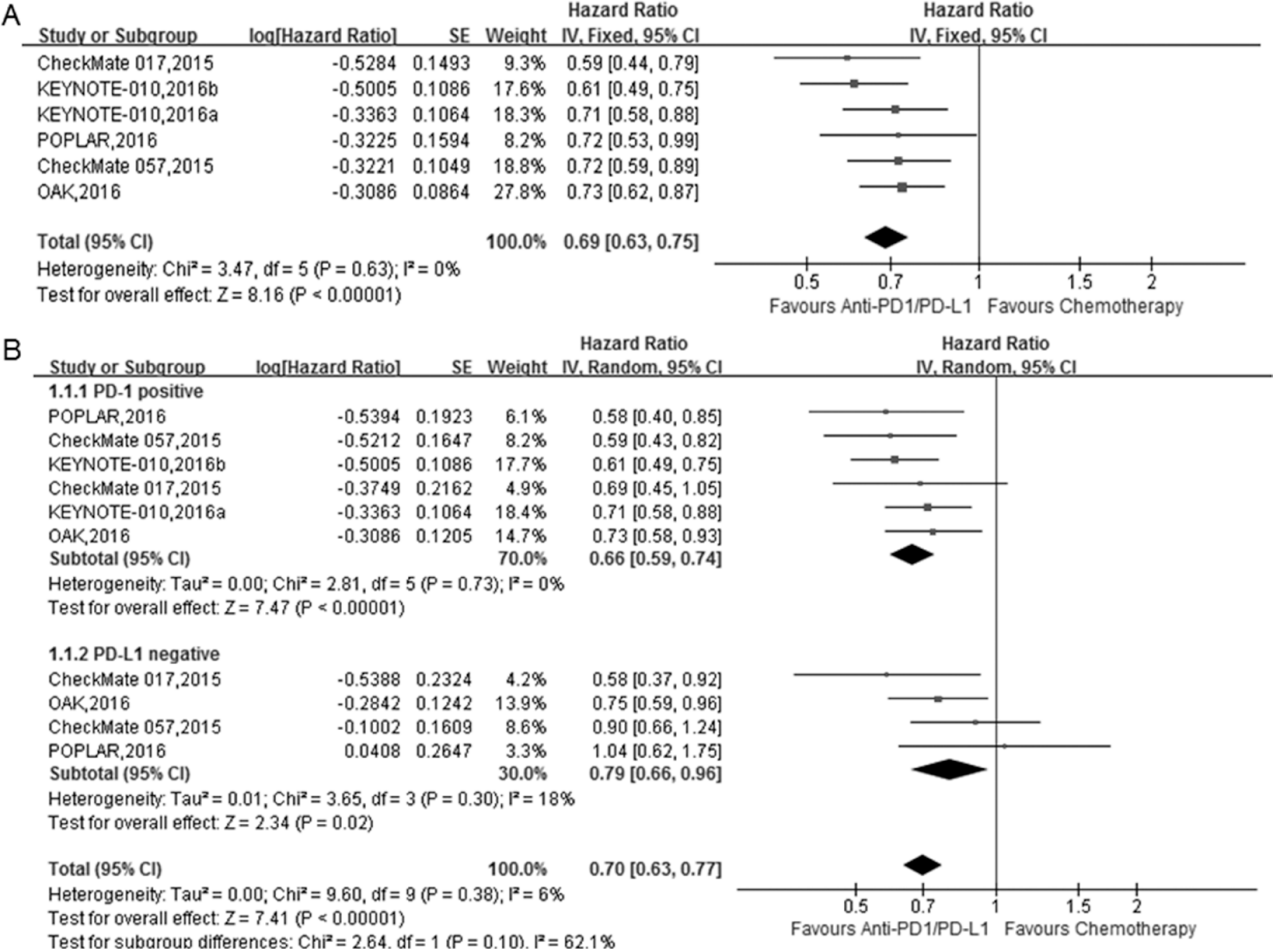
The forest plot of the overall survival (OS) in advanced NSCLC patients who received anti-PD1/PD-L1 antibody therapy compared to docetaxel (**A**) total; (**B**) subgroup analysis of OS based on PD-L1 expression level.

All trials reported the overall survival (OS) data. The median overall survival (OS) and the 95% confidence interval (95% CI), hazard ratio (HR) and the 95% CI for the treatment group versus control group were retrieved from the published edition as well as the supplementary materials (Table 2). The pooled HRs with 95% CIs for OS were calculated using the Review Manager 5.35. The pooled HR showed a significant improvement in OS for anti-PD-1/PD-L1 antibody therapy over docetaxel (Figure 2A; HR = 0.69, 95% CI: 0.63–0.75, *P* < 0.001).

**Table 2:** The OS and PFS in the 5 RCTs comparing anti-PD-1/anti-PD-L1 therapy with docetaxel

Name of RCTs	Study arms	Overall survival			Progression-free survival		
		Months (95% Cl)	Pooled HR (95% Cl)	*P* value	Months (95% Cl)	Pooled HR (95% Cl)	*P* value
POPLAR	Atezolizumab	12.6 (9.7–16.4)	0.73 (0.53–0.99)	*P* = 0.04	2.7 (2.0–4.1)	0.94 (0.72–1.23)	*P* = 0.645
	Docetaxel	9.7 (8.6–12.0)			3.0 (2.8–4.1)		
OAK	Atezolizumab	13.8 (11.8–15.7)	0.73 (0.62–0.87)	*P* = 0.0003	2.8 (2.6–3.0)	0.95 (0.82–1.1.0)	*P* = 0.49
	Docetaxel	9.6 (8.6–11.2)			4.0 (3.3–4.2)		
CheckMate 057	Nivolumab	12.2 (9.7–15.0)	0.73 (0.59–0.89)	*P* = 0.002	2.3 (2.2–3.3)	0.92 (0.77–1.11)	*P* = 0.39
	Docetaxel	9.4 (8.1–10.7)			4.2 (3.5–4.9)		
CheckMate 017	Nivolumab	9.2 (7.3–13.3)	0.59 (0.44–0.79)	p < 0.001	3.5 (2.1–4.9)	0.62 (0.47–0.81)	*p* < 0.001
	Docetaxel	6.0 (5.1–7.3)			2.8 (2.1–3.5)		
KEYNOTE-010	Pembrolizumab (2mg)	10.4 (9.4–11.9)	0.71 (0.58–0.88)	*P* = 0.0008	3.9 (3.1–4.1)	0.59 (0.44–0.78)	*P* = 0.0001
	Pembrolizumab (10mg)	12.7 (10.0–17.3)	0.61 (0.49–0.75)	*p* < 0.001	4.0 (2.7–4.3)	0.59 (0.45–0.78)	*p* < 0.001
	Docetaxel	8.5 (7.5–9.8)			4.0 (3.1–4.2)		

PD-L1 is a potential biomarker that is expressed on tumor cells and tumor-infiltrating immune cells. The PD-L1 expression level plays a crucial role in the prognosis of cancer patients [11, 17]. Therefore, we performed a subgroup analysis to assess the influence of PD-L1 expression level on the efficacy of anti-PD-1/PD-L1 antibody therapy. KEYNOTE-010 only enrolled patients whose biopsy and archives showed a PD-L1 tumor proportion score of 1% or greater (PD-L1 positive), but the remaining four RCTs included patients with different PD-L1 expression levels.

To better analyze the importance of PD-L1 expression, we redefined the positive PD-L1 as more than 1% or TC1/2/3 or IC1/2/3 based on the included 5 RCTs and analyzed the OS/PFS in the subgroups according to PD-L1 expression. We also defined the PD-L1 negative as less than 1% or TC0 and IC0.

The subgroup analysis according to PD-L1 expression level showed that in the PD-L1 positive subgroup, anti-PD-1/PD-L1 antibody therapy significantly improved the OS compared with docetaxel (Figure 2B; HR = 0.66, 95% CI 0.59–0.74, *P* < 0.001). In addition, for the PD-L1 negative subgroup, anti-PD-1/PD-L1 antibody therapy also significantly improved the OS compared with docetaxel (Figure 2B; HR = 0.79, 95% CI: 0.66–0.96, *P* = 0.02).

### Anti-PD-1/PD-L1 antibodies prolonged progression-free survival compared with docetaxel

The progression-free survival (PFS) remains controversial in several randomized clinical trials (Table 2). In the CheckMate-057, POPLAR and OAK studies, progression-free survival was similar between the treatment groups in the intention-to-treat population. However, in the CheckMate-017 and KEYNOTE-010 studies, PFS was improved after anti-PD1/PD-L1 antibody treatment, which showed superior efficacy to docetaxel. Thus, we calculated the pooled HRs for PFS in this study.

**Figure 3:**
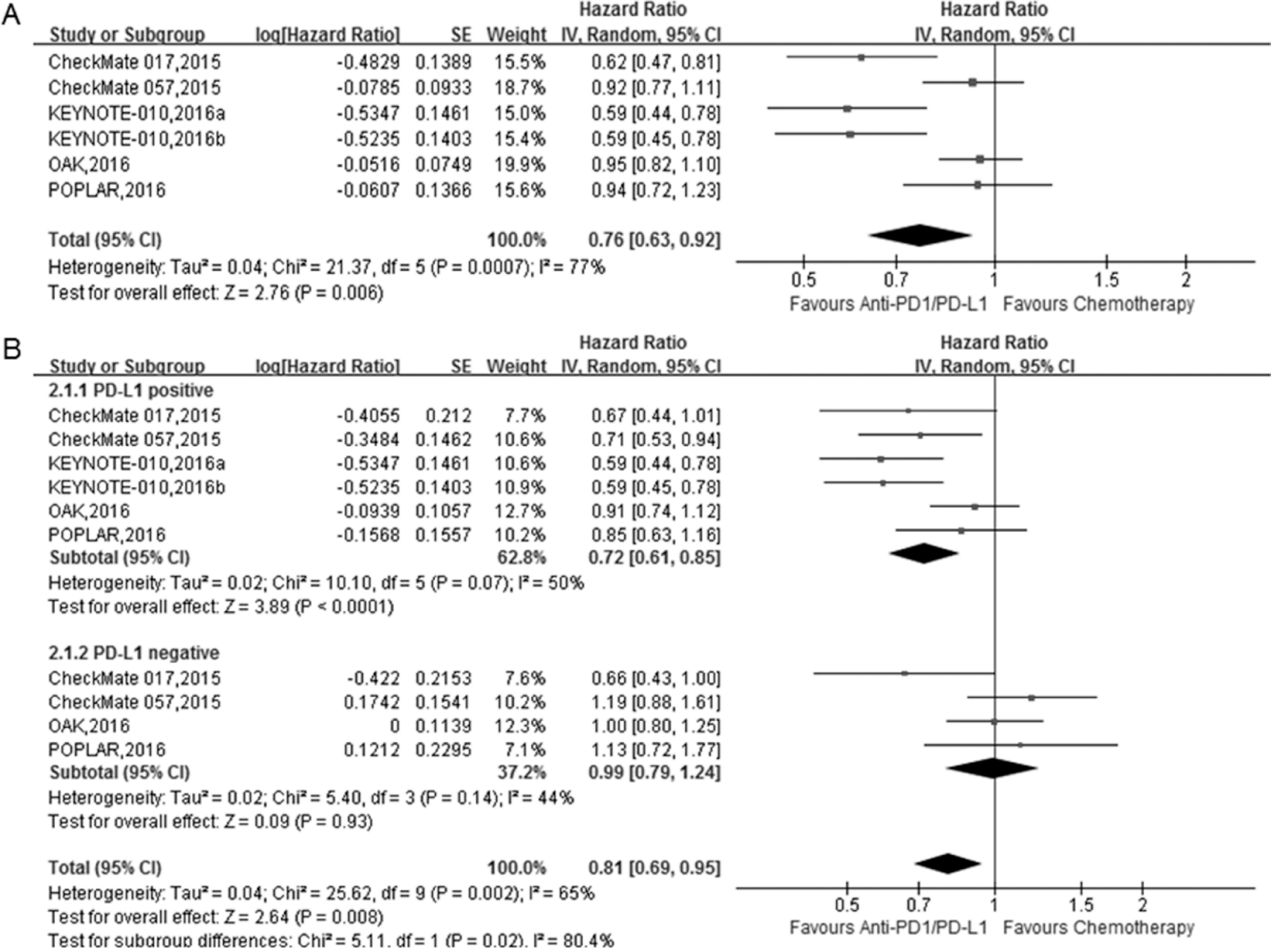
The forest plot of the progression-free survival (PFS) in advanced NSCLC patients who received anti-PD1/PD-L1 antibody therapy compared to docetaxel (**A**) total; (**B**) subgroup analysis of PFS based on PD-L1 expression level.

The pooled HRs showed a significant improvement in PFS for anti-PD-1/PD-L1 therapy compared with docetaxel (Figure 3A; HR = 0.76, 95% CI 0.63–0.92, *P* < 0.05).

The subgroup analysis based on the PD-L1 expression status showed that anti-PD1/PD-L1 antibody treatment improved PFS in the PD-L1 positive group (HR = 0.72, 95% CI 0.61–0.85, *P* < 0.001), but not in the PD-L1 negative group (Figure 3B; HR = 0.99, 95% CI 0.79–1.24, *P* = 0.93).

### Anti-PD-1/PD-L1 antibodies improved the objective response rate compared with docetaxel

**Figure 4:**
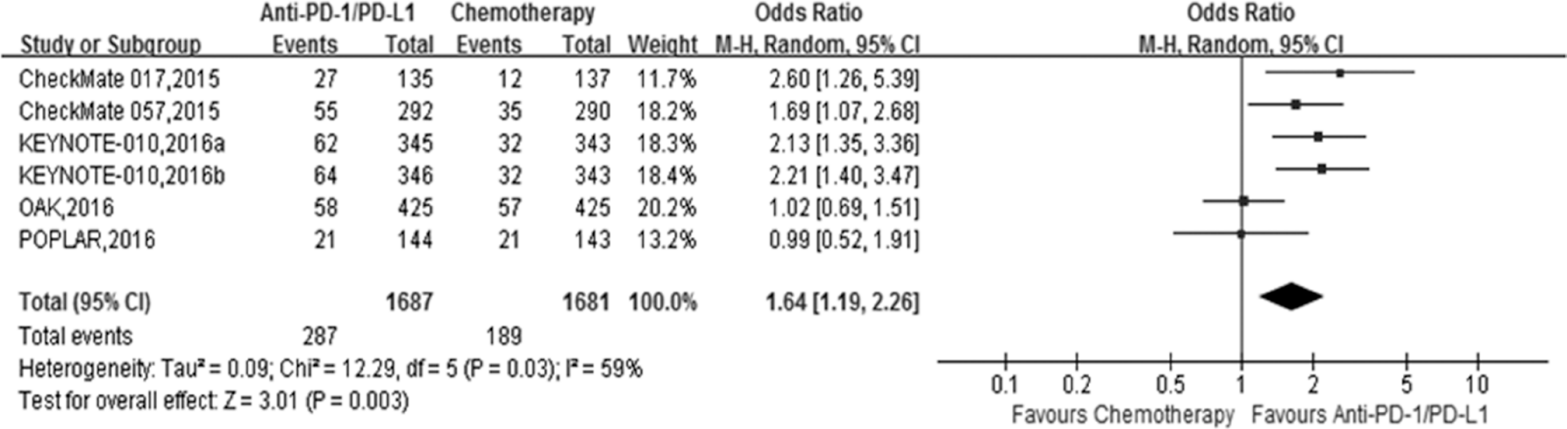
The forest plot of the objective response rate (ORR) in advanced NSCLC patients who received anti-PD1/PD-L1 antibody therapy compared to docetaxel

All the studies included in this meta-analysis reported the partial or complete overall response rate according to RECIST (version 1.1). We compared the overall response rate of anti-PD-1/PD-L1 antibodies (nivolumab, pembrolizumab and atezolizumab) with docetaxel for advanced NSCLC patients. The polled odds ratio (OR) for overall response rate (ORR) was 1.64 (Figure 4; 95% CI 1.19–2.26, *P* < 0.05), which suggested a higher clinical response rate for anti-PD-1/PD-L1 antibodies than for docetaxel in advanced NSCLC patients.

### Anti-PD-1/PD-L1 antibodies showed lower toxicity than docetaxel

All studies included in this meta-analysis reported treatment-related adverse events (Table 3), as well as treatment-related grade 3 or 4 adverse events according to the National Cancer Institute Common Terminology Criteria for Adverse Events version 4.0. Adverse events were listed in both treatment arms, such as fatigue, decreased appetite, nausea, diarrhea, anemia, pneumonitis, hyperthyroidism, and hypothyroidism,. The pooled ORs showed significantly lower rates of any grade adverse events in the anti-PD-1/PD-L1 groups than in the docetaxel groups, including alopecia (OR = 0.01, 95% CI 0.01–0.02), fatigue (OR = 0.54, 95% CI 0.46–0.63), nausea (OR = 0.52, 95% CI 0.39–0.70), diarrhea (OR = 0.36, 95% CI 0.27–0.49), anemia (OR = 0.21, 95% CI 0.12–0.37), decreased appetite (OR = 0.78, 95% CI 0.60–1.00), and neutropenia (OR = 0.04, 95% CI 0.02–0.06) (Table 4, Supplementary Figures). Hyperthyroidism and hypothyroidism are immune-mediated adverse events, and the pooled ORs showed that the risk of these two AEs was significantly higher in the anti-PD-1/PD-L1 groups (Table 4, Supplementary Figures).

**Table 3:** The adverse events in the 5 RCTs comparing anti-PD-1/anti-PD-L1 therapy with docetaxel

Name of RCTs	Study arms	No. of patients	Treatment-related AEs	Treatment-related AEs Grade ≥3
POPLAR	Atezolizumab	144	67% (95/142)	12% (17/142)
	Docetaxel	143	88% (119/135)	41% (55/135)
OAK	Atezolizumab	425	64% (390/609)	15% (90/609)
	Docetaxel	425	86% (496/578)	43% (247/578)
CheckMate 057	Nivolumab	292	69% (199/287)	10% (30/287)
	Docetaxel	290	88% (236/268)	54% (144/268)
CheckMate 017	Nivolumab	135	58% (76/131)	7% (9/131)
	Docetaxel	137	86% (111/129)	55% (71/129)
KEYNOTE-010	Pembrolizumab (2 mg)	345	63% (215/339)	13% (43/339)
	Pembrolizumab (10 mg)	346	66% (226/343)	16% (55/343)
	Docetaxel	343	81% (251/309)	35% (109/309)

**Table 4:** Comparative adverse events (any grade) of anti-PD-1/PD-L1 group versus docetaxel group in RCTs

Adverse Events	No. of trials	*P* group events/pts	D group events/pts	Pooled OR (95% CI)	*P* value
Alopecia	5	11/1851	551/1728	0.01 (0.01,0.02)	*P* < 0.001
Fatigue	5	354/1851	524/1728	0.54 (0.46,0.63)	*P* < 0.001
Nausea	5	239/1851	358/1728	0.52 (0.39,0.70)	*P* < 0.001
Diarrhea	5	182/1851	371/1728	0.36 (0.27,0.49)	*P* < 0.001
Anemia	5	110/1851	319/1728	0.21 (0.12,0.37)	*P* < 0.001
Decreased appetite	5	291/1851	322/1728	0.78 (0.60,1.00)	*P* = 0.05
Asthenia	5	206/1851	267/1728	0.60 (0.44,0.83)	*P* = 0.002
Vomiting	3	107/1433	126/1331	0.61 (0.33,1.10)	*P* = 0.10
Neutropenia	5	15/1851	318/1728	0.04 (0.02,0.06)	*P* < 0.001
Hyperthyroidism	1	25/682	0/618	24.44 (3.31, 180.61)	*P* < 0.05
Hypothyroidism	1	48/682	2/618	23.32 (5.64, 96.37)	*P* < 0.001

*P* group: anti-PD-1/PD-L1 group; D group: docetaxel group; pts: patients.

**Figure 5:**
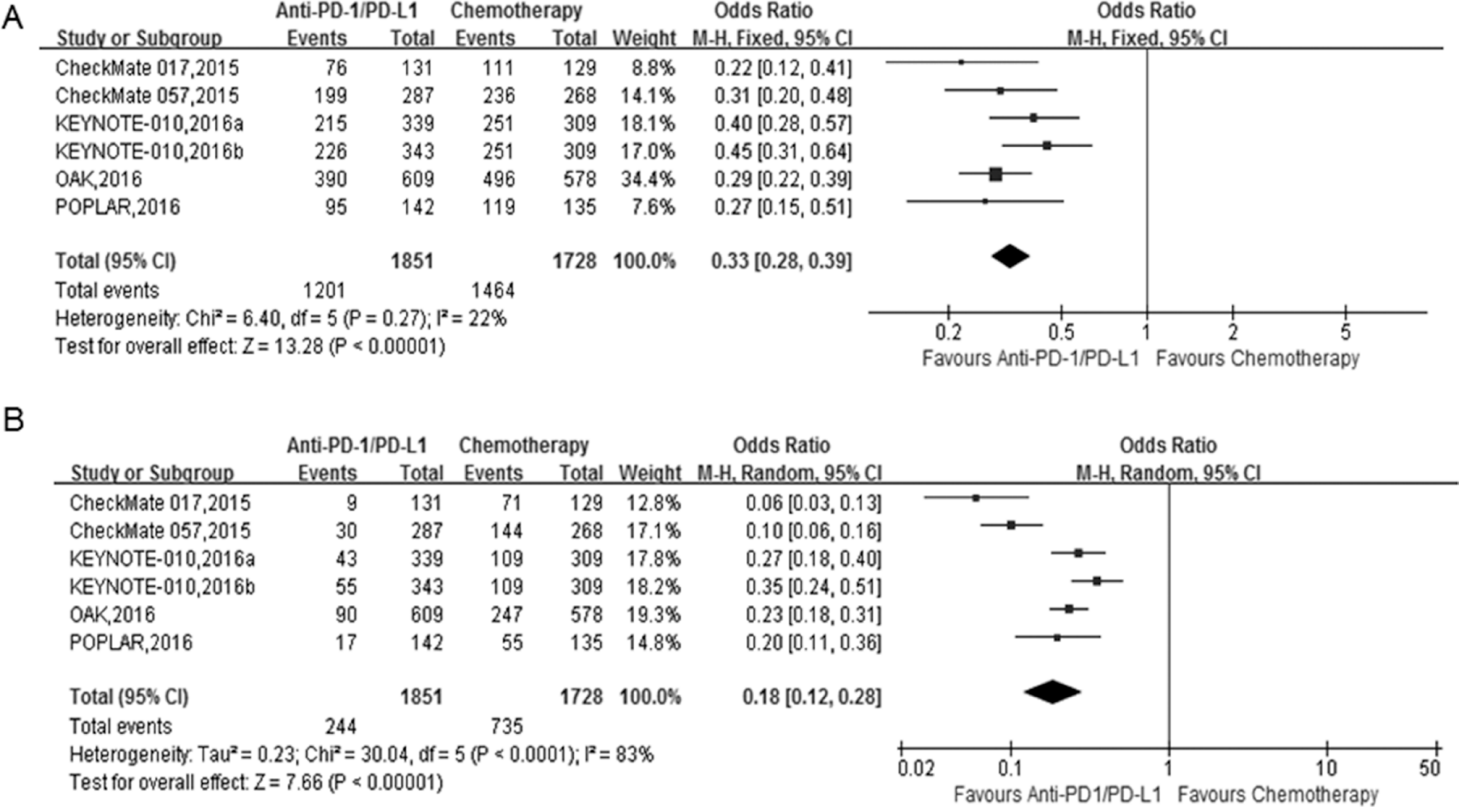
The forest plot of the adverse events (AEs) in advanced NSCLC patients who received anti-PD1/PD-L1 antibody therapy compared to docetaxel (**A**) treatment-related AEs; (**B**) severe treatment-related AEs (Grade ≥ 3).

Anti-PD1/PD-L1 antibody therapy showed significantly lower toxicity than chemotherapy for treatment-related adverse events (Figure 5A; OR = 0.33, 95% CI 0.28–0.39, *P* < 0.001). Moreover, we compared the severe adverse events (grade 3,4, or 5) of anti-PD-1/PD-L1 antibody therapy with chemotherapy for advanced NSCLC patients. In this analysis, the anti-PD-1/PD-L1 antibody therapy also showed significantly lower toxicity than the chemotherapy groups (Figure 5B; OR = 0.18, 95% CI 0.12–0.28, *P* < 0.001).

## DISCUSSION

Programmed death protein 1 (PD-1) is a co-inhibitory molecule expressed by activated T cells. When it binds its ligands, PD-L1 or PD-L2, T-cell activation is inhibited and antitumor immune response is dampened [18, 19]. PD-L1 is expressed on tumor cells as well as tumor-infiltrating T cells in many kinds of cancers. Therefore, the PD-1/PD-L1 pathway plays an important role in tumor immunologic escape [8].

In recent years, antibodies targeting the PD-1/PD-L1 pathway have been widely explored in clinical trials and have exhibited satisfactory results [20]. Nivolumab, an IgG4 monoclonal antibody that targets the PD-1 receptor, is now being used in the clinical trials of non-small-cell lung cancer, metastatic melanoma, renal-cell carcinoma [21], ovarian cancer and Hodgkin’s lymphoma [9]. It was approved in 2015 by the Food and Drug Administration (FDA) for the treatment of previously treated advanced or metastatic NSCLC 5 [22]. Pembrolizumab is an IgG4-engineered humanized antibody that targets the PD-1 receptor. It is now being used in clinical trials for advanced melanoma, advanced urothelial cancer, and NSCLC. The US FDA granted accelerated approval to pembrolizumab for the treatment of metastatic NSCLC patients whose tumors expressed high levels of PD-L1 [22, 23]. Atezolizumab (MPDL3280A) is a humanized engineered IgG1 monoclonal antibody against PD-L1. Several clinical trials have also been designed to evaluate the efficacy and safety of atezolizumab in the treatment of many tumors, including NSCLC [13, 16].

These PD-1/PD-L1 inhibitors are breakthroughs in the treatment of NSCLC [23]. Some clinical trials proved the safety and efficacy of anti-PD-1/PD-L1 antibodies, and other studies compared the treatment effects of PD-1/PD-L1 inhibitors therapy and chemotherapy. A few meta-analyses on PD-1/PD-L1 inhibitors in the treatment of NSCLC patients have been published. For example, Jiaxing Huang *et al.* [7] wrote a meta-analysis about the efficacy and safety of PD-1 inhibitors in previously treated advanced NSCLC patients. However, this study only enrolled clinical trials involving nivolumab, and most of the trials included were single-arm treatments without a control group. Guo-Wu Zhou *et al.* [18] conducted a similar meta-analysis comparing anti-PD1/PD-L1 antibody therapy with chemotherapy for pretreated NSCLC patients, but they only included three randomized clinical trials enrolling 1141 patients who received treatment with nivolumab or atezolizumab. Additionally, because of the time of publication, recently published high-quality literature was not included.

Some phase II/III clinical trials published recently provided more information about the safety and efficacy of anti-PD-1/PD-L1 antibody therapy [13, 16]. In our meta-analysis, we included 5 randomized clinical trials to evaluate the efficacy and safety of anti-PD-1/PD-L1 antibody therapy compared with docetaxel in previously treated advanced NSCLC patients.

In these clinical trials, all patients with stage IIIB or IV NSCLC had previous treatment, such as surgical resection, radiation therapy or platinum-based chemotherapy, and these patients had tumor recurrence or progression during or after the regular treatment. All patients enrolled in the experimental groups received the anti-PD-1/PD-L1 antibodies intravenously at an appropriate dose identified by the previously conducted phase I clinical trials. In the control groups, the participants received docetaxel intravenously at a dose of 75 mg/m^2^. The expression of PD-L1 in tumor specimens was detected by immunohistochemistry (IHC). All clinical trials were conducted under the guidance of previously designed protocols, and all participants were followed up regularly during the clinical trials.

Our meta-analysis demonstrated that immune checkpoint inhibitors significantly improved efficacy in previously treated advanced NSCLC patients using OS/PFS/ORR as the primary or secondary endpoints.

PD-L1 is a potential biomarker for anti-PD1/PD-L1 antibodies; a positive status is defined differently in these clinical trials. In CheckMate-017 and CheckMate-057, more than 1% of positive IHC staining cancer cells were defined as PD-L1 positive. The KEYNOTE-010 clinical trial only enrolled patients with PD-L1 expression ≥1%. In POPLAR and OAK, IHC staining of PD-L1 expression was detected on both tumor cells (TC) and tumor-infiltrating immune cells (IC). TC1/2/3 or IC1/2/3 was defined as PD-L1 positive, and TC0 and IC0 were defined as PD-L1 negative. To better reflect the role of PD-L1 expression in PD1/PD-L1 inhibitors treatment, we redefined the positive PD-L1 as more than 1% or TC1/2/3 or IC1/2/3 based on the included 5 RCTs and analyzed the OS/PFS in the subgroups according to PD-L1 expression. In the PD-L1 positive subgroup, anti-PD-1/PD-L1 antibody therapy showed significantly improved OS compared with chemotherapy (*P* < 0.001) and significantly prolonged PFS (*P* < 0.001). However, the improvement of OS between the two treatments in the PD-L1 negative subgroup (*P* = 0.02) was not as much as that in the PD-L1 positive subgroup (*P* < 0.001), and there was no significant difference in PFS between the two groups (*P* > 0.05). We found that PD-L1 expression might be an important prognostic factor for the efficacy of PD-1/PD-L1 inhibitors in advanced NSCLC. However, we did not compare objective response rate (ORR) or adverse events (AEs) in the subgroup according to PD-L1 expression due to the lack of data.

Consistent with previous findings in clinical trials of different phases, our study demonstrated a more favorable safety profile for PD-1/PD-L1 inhibitors than that of second-line docetaxel chemotherapy. Treatment-related adverse events and severe adverse events (grade ≥3) including fatigue, decreased appetite, nausea, diarrhea, and anemia were identified in all trials. The side effects of anti-PD1/PD-L1 antibody therapy were less than the docetaxel groups. This finding might be related to the damage of epithelium-derived cells and renewing cell populations caused by docetaxel. Although anti-PD1/PD-L1 antibodies caused few chemotherapy-related adverse events, the immune-mediated adverse events, including inflammatory pneumonitis, interstitial nephritis, hyperthyroidism, and hypothyroidism, occurred more frequently in pulmonary, endocrine, mucocutaneous and renal sites and even immunologically privileged sites such as the eye. Most of these immune-mediated adverse events were moderate and could be controlled by following guidelines. Occasionally, the side effects were life threatening, such as severe inflammatory pneumonitis, and required cessation of therapy and treatment with immunosuppressants such as corticosteroids [24]. It was rare that severe toxic events led to the discontinuation of treatment or death of a patient. Therefore, the immune-mediated adverse events were relatively tolerable and acceptable. Our study demonstrates that PD1/PD-L1 antibody therapy is safer and more effective than docetaxel, which supports future clinical applications of anti-PD-1/PD-L1 antibody-based immunotherapy.

However, our study has some limitations. First, we extracted data from published articles without individual patient data, which might result in the bias of data analysis. Second, the definition of PD-L1 expression on the tumor and tumor-infiltrating cells remains inconsistent in different clinical trials. For this reason, we formulated a uniform definition of PD-L1 expression in patients within all these clinical trials. Third, we only included RCTs using docetaxel because it is the most common drug used as the second line of chemotherapy in advanced NSCLC. Therefore, the number of studies included in this meta-analysis is small. Because of the above limitations in our study, further studies based on the information from ongoing trials are needed to verify the efficacy and safety of anti-PD1/PD-L1 therapy versus docetaxel in patients with advanced NSCLC.

In conclusion, our study indicates that anti-PD-1/PD-L1 antibody therapy improves PFS, OS and ORR and shows less toxicity in patients with advanced or metastatic NSCLC. Despite some limitations, our study suggests that immune checkpoint inhibitors may provide a promising therapeutic strategy for patients with advanced NSCLC.

## METHODS

### Literature search strategy

We searched relevant databases to select corresponding clinical trials, such as Pubmed (Medline), EMBASE, the Corane library, clinicaltrial.gov, and ASCO meeting abstracts (until April 20, 2017). The following terms were used to select trial publications or presentations: non-small cell lung cancer, NSCLC, nivolumab, pembrolizumab, atezolizumab, PD-1, PD-L1, immunotherapy, and randomized clinical trial. We searched for publications and unpublished trials written in the English language.

### Inclusion and exclusion criteria

The eligible literature was confined to randomized clinical trials written in English. The studies included met the following criteria: (1) Published studies comparing anti-PD-1/PD-L1 antibodies with docetaxel for patients with pretreated advanced non-small cell lung cancer; (2) The outcomes of the trials were available: overall survival (OS), progression-free-survival (PFS), objective response rate (ORR), adverse events (AE), and hazard ratio (HR). The exclusion criteria were (1) The phase I trials and (2) studies with no available outcomes.

### Data extraction and quality assessment

Two investigators conducted the literature research and reviewed the studies independently to avoid bias. Disagreements were resolved by discussion and adjudicated by a third investigator. For the included studies, we extracted the following data: authors, year of publication, abbreviations of the trials, registered number, trial phase, dose of drugs, number of enrolled patients, tumor histology, PD-L1 expression level, as well as the outcomes mentioned above.

The quality of the studies included was assessed using the method reported by Jadad *et al* [25]. We scored the papers and answered the following questions: (1) Was the study described as randomized? (0–2 points); (2) Was the study described as blinded? (0–2 points); and (3) Was there a description of withdrawals and dropouts? (0–1 point). If the trial scored fewer than 3 points, it was considered to be low quality. Trials that scored ≥3 points were considered to be high quality.

### Statistical analysis

We used the Review Manager 5.3.5 to perform the statistical analysis under the guidance of the Cochrane library. The pooled HRs (hazard ratio) with 95% CIs for OS and PFS, and the ORs (odds ratio) with 95% CIs for ORR and AEs were calculated using the Review Manager 5.35. HRs > 1 favored the docetaxel arm while HRs < 1 favored the anti-PD-1/PD-L1 antibodies arm. ORs > 1 for ORR and AEs meant a higher response rate and toxicity, whereas ORs < 1 reflected lower response rate and safety. *P* < 0.05 was considered to be statistically significant. The I^2^ statistic and Q statistics were used to test statistical heterogeneity of included studies, with a predefined significant threshold of *I*^2^ < 50% or *p* > 0.1. If the *I*^2^ was ≤ 50%, then the trials were considered to be homogeneous, and a fixed-effect model was used. Otherwise, a random-effect model was used.

## SUPPLEMENTARY MATERIALS FIGURES


