# Supplementary material for: The efficacy and safety of anti-PD-1/PD-L1 antibody therapy versus docetaxel for pretreated advanced NSCLC: a meta-analysis
Source: Oncotarget. 2017 Dec 15;9(3):4239–48. doi: 10.18632/oncotarget.23279 (PMC5790535; doi:10.18632/oncotarget.23279)
Supplement: Supplementary file 1 [file oncotarget-09-4239-s001.pdf]

The efficacy and safety of anti-PD-1/PD-L1 antibody therapy versus docetaxel for pretreated advanced NSCLC: a meta-analysis

SUPPLEMENTARY MATERIALS

Study or Subgroup	Experimental Events	Experimental Total	Control Events	Control Total	Weight	Odds Ratio M-H, Fixed, 95% CI
CheckMate 017,2015	0	131	29	129	5.2%	0.01 [0.00, 0.21]
CheckMate 057,2015	1	287	67	268	12.2%	0.01 [0.00, 0.08]
KEYNOTE-010,2016a	3	339	101	309	18.5%	0.02 [0.01, 0.06]
KEYNOTE-010,2016b	2	343	101	309	18.6%	0.01 [0.00, 0.05]
OAK,2016	3	609	202	578	36.4%	0.01 [0.00, 0.03]
POPLAR,2016	2	142	51	135	9.1%	0.02 [0.01, 0.10]
**Total (95% CI)**		**1851**		**1728**	**100.0%**	**0.01 [0.01, 0.02]**
Total events	11		551			

Heterogeneity: Chi² = 1.39, df = 5 (P = 0.93); I² = 0%
Test for overall effect: Z = 14.38 (P < 0.00001)

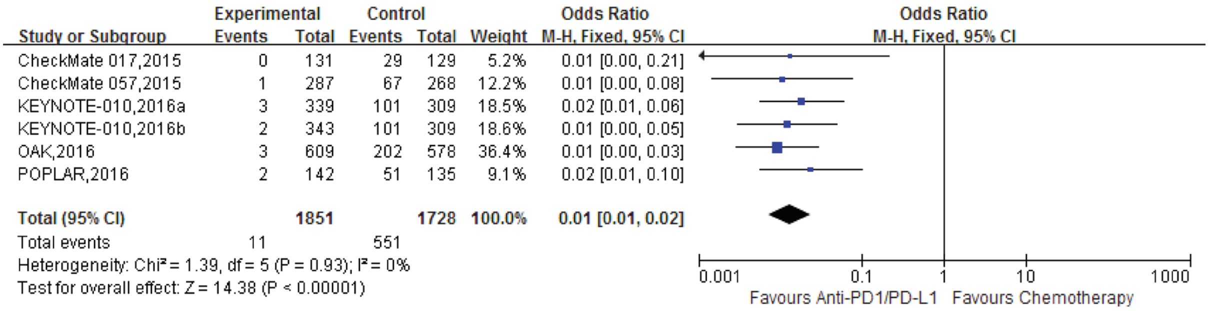

Supplementary Figure 1: The forest plot of alopecia in advanced NSCLC patients who received anti-PD1/PD-L1 antibody therapy compared to docetaxel.

Study or Subgroup	Anti-PD1/PD-L1 Events	Anti-PD1/PD-L1 Total	Chemotherapy Events	Chemotherapy Total	Weight	Odds Ratio M-H, Fixed, 95% CI
CheckMate 017,2015	21	131	42	129	8.2%	0.40 [0.22, 0.72]
CheckMate 057,2015	46	287	78	268	15.6%	0.46 [0.31, 0.70]
KEYNOTE-010,2016a	46	339	76	309	15.9%	0.48 [0.32, 0.72]
KEYNOTE-010,2016b	49	343	76	309	15.8%	0.51 [0.34, 0.76]
OAK,2016	163	609	205	578	35.6%	0.66 [0.52, 0.85]
POPLAR,2016	29	142	47	135	8.9%	0.48 [0.28, 0.82]
**Total (95% CI)**		**1851**		**1728**	**100.0%**	**0.54 [0.46, 0.63]**
Total events	354		524			

Heterogeneity: Chi² = 4.84, df = 5 (P = 0.44); I² = 0%
Test for overall effect: Z = 7.71 (P < 0.00001)

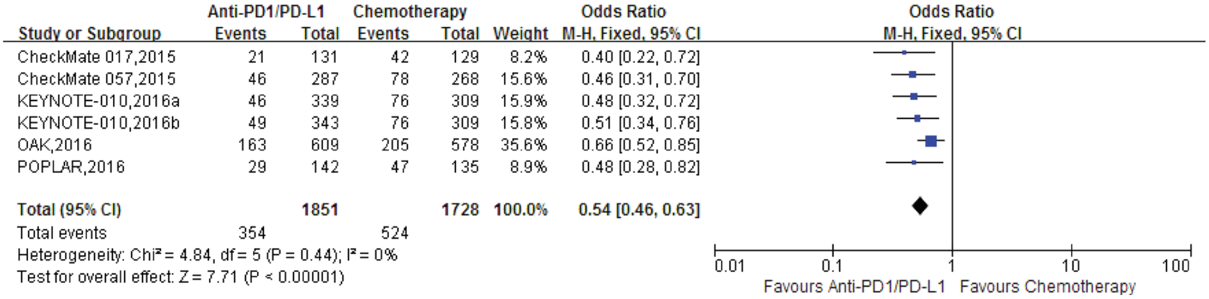

Supplementary Figure 2: The forest plot of fatigue in advanced NSCLC patients who received anti-PD1/PD-L1 antibody therapy compared to docetaxel.

Study or Subgroup	Anti-PD1/PD-L1 Events	Anti-PD1/PD-L1 Total	Chemotherapy Events	Chemotherapy Total	Weight	Odds Ratio M-H, Random, 95% CI
CheckMate 017,2015	12	131	30	129	10.8%	0.33 [0.16, 0.68]
CheckMate 057,2015	34	287	70	268	18.0%	0.38 [0.24, 0.60]
KEYNOTE-010,2016a	37	339	45	309	17.5%	0.72 [0.45, 1.14]
KEYNOTE-010,2016b	31	343	45	309	16.8%	0.58 [0.36, 0.95]
OAK,2016	108	609	131	578	24.2%	0.74 [0.55, 0.98]
POPLAR,2016	17	142	37	135	12.7%	0.36 [0.19, 0.68]
**Total (95% CI)**		**1851**		**1728**	**100.0%**	**0.52 [0.39, 0.70]**
Total events	239		358			

Heterogeneity: Tau² = 0.07; Chi² = 11.33, df = 5 (P = 0.05); I² = 56%
Test for overall effect: Z = 4.32 (P < 0.0001)

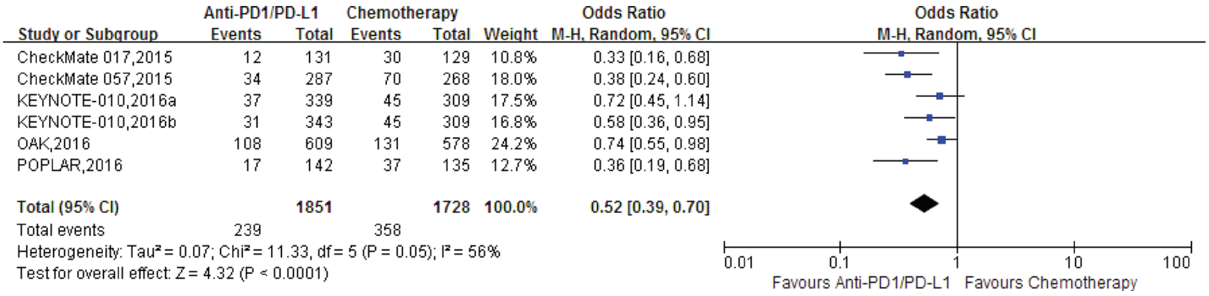

Supplementary Figure 3: The forest plot of nausea in advanced NSCLC patients who received anti-PD1/PD-L1 antibody therapy compared to docetaxel.

Study or Subgroup	Anti-PD1/PD-L1 Events	Anti-PD1/PD-L1 Total	Chemotherapy Events	Chemotherapy Total	Weight	Odds Ratio M-H, Random, 95% CI
CheckMate 017,2015	10	131	26	129	10.6%	0.33 [0.15, 0.71]
CheckMate 057,2015	22	287	62	268	17.2%	0.28 [0.16, 0.46]
KEYNOTE-010,2016a	24	339	56	309	17.7%	0.34 [0.21, 0.57]
KEYNOTE-010,2016b	22	343	56	309	17.2%	0.31 [0.18, 0.52]
OAK,2016	94	609	141	578	26.4%	0.57 [0.42, 0.76]
POPLAR,2016	10	142	30	135	10.9%	0.27 [0.12, 0.57]
**Total (95% CI)**		**1851**		**1728**	**100.0%**	**0.36 [0.27, 0.49]**
Total events	182		371			

Heterogeneity: Tau² = 0.07; Chi² = 10.10, df = 5 (P = 0.07); I² = 50%
Test for overall effect: Z = 6.64 (P < 0.00001)

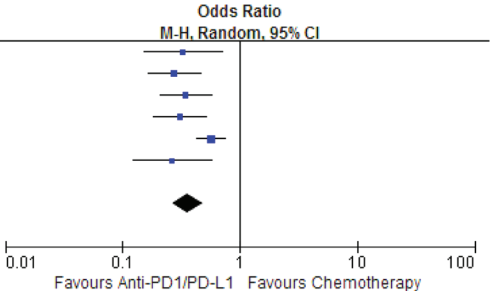

Supplementary Figure 4: The forest plot of diarrhea in advanced NSCLC patients who received anti-PD1/PD-L1 antibody therapy compared to docetaxel.

Study or Subgroup	Anti-PD1/PD-L1 Events	Anti-PD1/PD-L1 Total	Chemotherapy Events	Chemotherapy Total	Weight	Odds Ratio M-H, Random, 95% CI
CheckMate 017,2015	2	131	28	129	9.2%	0.06 [0.01, 0.24]
CheckMate 057,2015	6	287	53	268	15.6%	0.09 [0.04, 0.21]
KEYNOTE-010,2016a	10	339	40	309	17.7%	0.20 [0.10, 0.42]
KEYNOTE-010,2016b	14	343	40	309	18.8%	0.29 [0.15, 0.54]
OAK,2016	70	609	136	578	22.9%	0.42 [0.31, 0.58]
POPLAR,2016	8	142	22	135	15.8%	0.31 [0.13, 0.72]
**Total (95% CI)**		**1851**		**1728**	**100.0%**	**0.21 [0.12, 0.37]**
Total events	110		319			

Heterogeneity: Tau² = 0.33; Chi² = 19.31, df = 5 (P = 0.002); I² = 74%
Test for overall effect: Z = 5.42 (P < 0.00001)

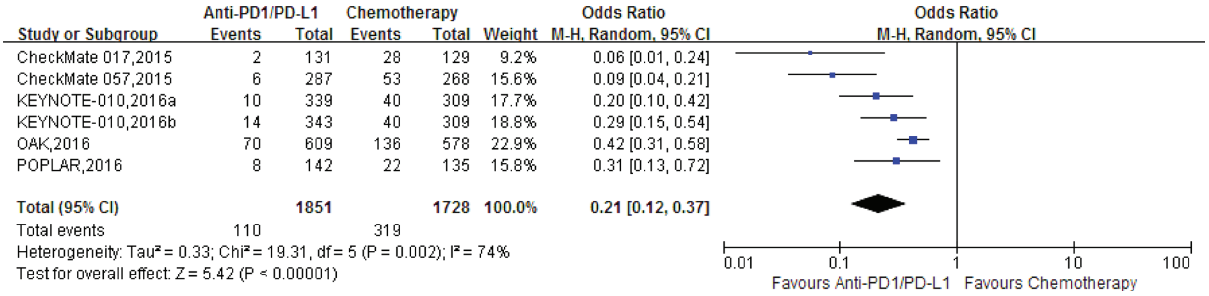

Supplementary Figure 5: The forest plot of anemia in advanced NSCLC patients who received anti-PD1/PD-L1 antibody therapy compared to docetaxel.

Study or Subgroup	Experimental Events	Experimental Total	Control Events	Control Total	Weight	Odds Ratio M-H, Random, 95% CI
CheckMate 017,2015	14	131	25	129	9.7%	0.50 [0.25, 1.01]
CheckMate 057,2015	30	287	42	268	15.6%	0.63 [0.38, 1.04]
KEYNOTE-010,2016a	46	339	49	309	18.4%	0.83 [0.54, 1.29]
KEYNOTE-010,2016b	33	343	49	309	16.8%	0.56 [0.35, 0.90]
OAK,2016	143	609	136	578	28.1%	1.00 [0.76, 1.30]
POPLAR,2016	25	142	21	135	11.3%	1.16 [0.61, 2.19]
**Total (95% CI)**		**1851**		**1728**	**100.0%**	**0.78 [0.60, 1.00]**
Total events	291		322			

Heterogeneity: Tau² = 0.04; Chi² = 8.61, df = 5 (P = 0.13); I² = 42%
Test for overall effect: Z = 1.98 (P = 0.05)

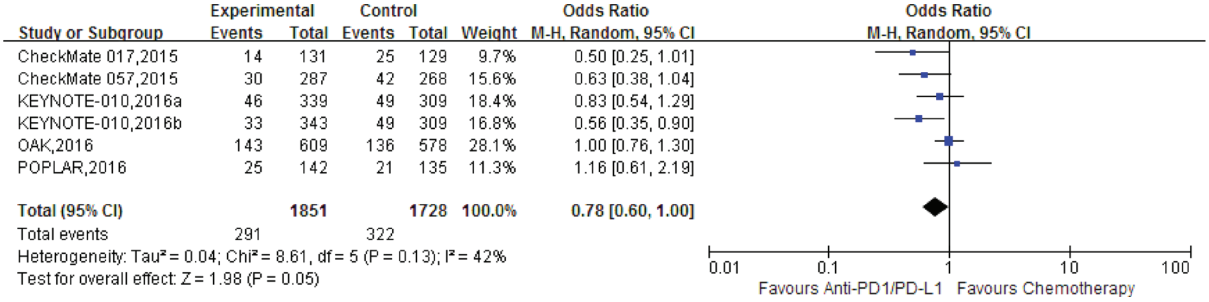

Supplementary Figure 6: The forest plot of decreased appetite in advanced NSCLC patients who received anti-PD1/PD-L1 antibody therapy compared to docetaxel.

Study or Subgroup	Experimental Events	Experimental Total	Control Events	Control Total	Weight	Odds Ratio M-H, Random, 95% CI
CheckMate 017,2015	13	131	18	129	11.7%	0.68 [0.32, 1.45]
CheckMate 057,2015	29	287	47	268	18.8%	0.53 [0.32, 0.87]
KEYNOTE-010,2016a	20	339	35	309	16.4%	0.49 [0.28, 0.87]
KEYNOTE-010,2016b	19	343	35	309	16.1%	0.46 [0.26, 0.82]
OAK,2016	116	609	114	578	26.7%	0.96 [0.72, 1.28]
POPLAR,2016	9	142	18	135	10.3%	0.44 [0.19, 1.02]
**Total (95% CI)**		**1851**		**1728**	**100.0%**	**0.60 [0.44, 0.83]**
Total events	206		267			

Heterogeneity: Tau² = 0.08; Chi² = 10.45, df = 5 (P = 0.06); I² = 52%
Test for overall effect: Z = 3.07 (P = 0.002)

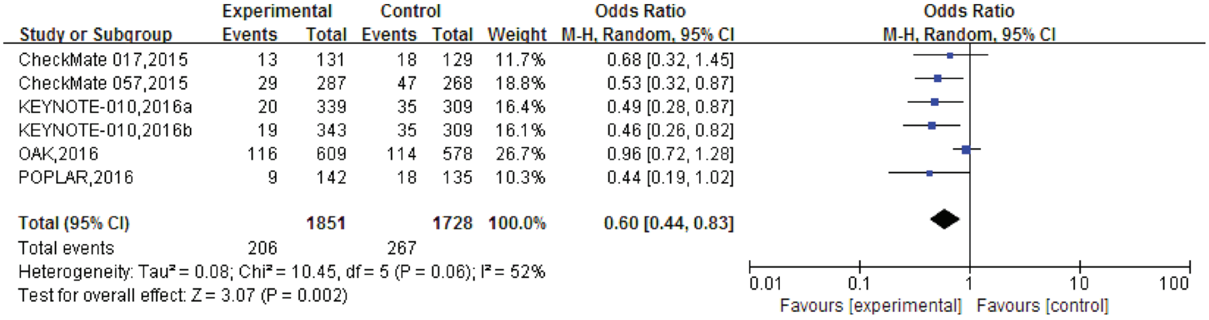

Supplementary Figure 7: The forest plot of asthenia in advanced NSCLC patients who received anti-PD1/PD-L1 antibody therapy compared to docetaxel.

Study or Subgroup	Anti-PD1/PD-L1 Events	Anti-PD1/PD-L1 Total	Chemotherapy Events	Chemotherapy Total	Weight	Odds Ratio M-H, Random, 95% CI
KEYNOTE-010,2016a	12	339	24	309	23.8%	0.44 [0.21, 0.89]
KEYNOTE-010,2016b	13	343	24	309	24.2%	0.47 [0.23, 0.94]
OAK,2016	74	609	62	578	31.8%	1.15 [0.80, 1.65]
POPLAR,2016	8	142	16	135	20.1%	0.44 [0.18, 1.07]
**Total (95% CI)**		**1433**		**1331**	**100.0%**	**0.61 [0.33, 1.10]**
Total events	107		126			

Heterogeneity: Tau² = 0.26; Chi² = 10.76, df = 3 (P = 0.01); I² = 72%
Test for overall effect: Z = 1.64 (P = 0.10)

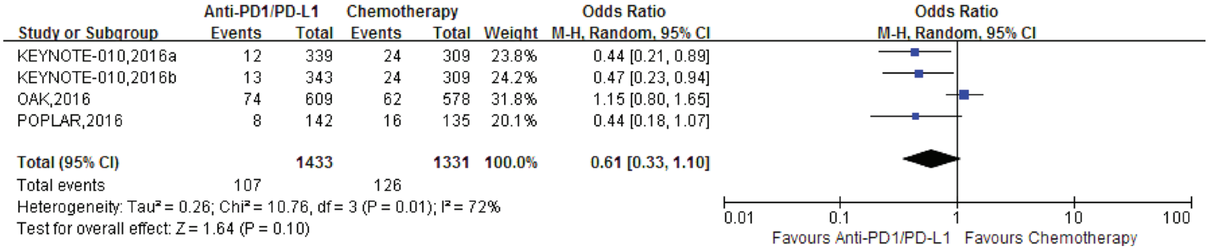

Supplementary Figure 8: The forest plot of vomiting in advanced NSCLC patients who received anti-PD1/PD-L1 antibody therapy compared to docetaxel.

Study or Subgroup	Anti-PD1/PD-L1 Events	Anti-PD1/PD-L1 Total	Chemotherapy Events	Chemotherapy Total	Weight	Odds Ratio M-H, Fixed, 95% CI
CheckMate 017,2015	1	131	42	129	12.9%	0.02 [0.00, 0.12]
CheckMate 057,2015	1	287	83	268	26.3%	0.01 [0.00, 0.06]
KEYNOTE-010,2016a	1	339	44	309	14.1%	0.02 [0.00, 0.13]
KEYNOTE-010,2016b	1	343	44	309	14.2%	0.02 [0.00, 0.13]
OAK,2016	10	609	90	578	27.9%	0.09 [0.05, 0.18]
POPLAR,2016	1	142	15	135	4.7%	0.06 [0.01, 0.44]
**Total (95% CI)**		**1851**		**1728**	**100.0%**	**0.04 [0.02, 0.06]**
Total events	15		318			

Heterogeneity: Chi² = 11.25, df = 5 (P = 0.05); I² = 56%
Test for overall effect: Z = 12.49 (P < 0.00001)

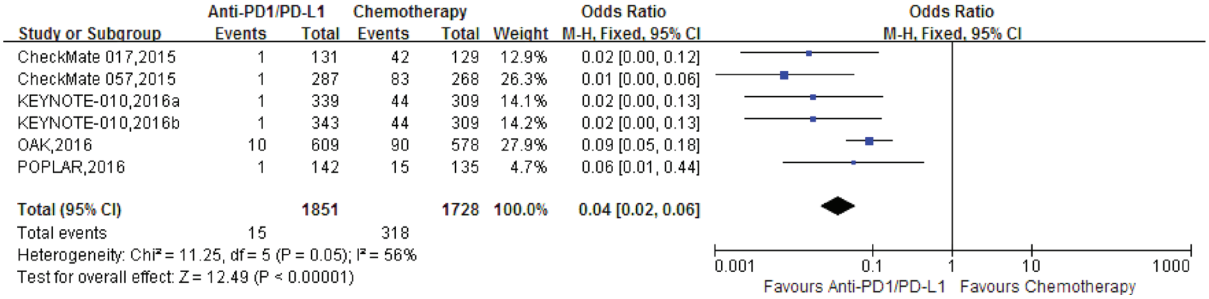

Supplementary Figure 9: The forest plot of neutropenia in advanced NSCLC patients who received anti-PD1/PD-L1 antibody therapy compared to docetaxel.

Study or Subgroup	Anti-PD1/PD-L1 Events	Anti-PD1/PD-L1 Total	Chemotherapy Events	Chemotherapy Total	Weight	Odds Ratio M-H, Fixed, 95% CI
KEYNOTE-010,2016a	10	339	0	309	50.2%	19.73 [1.15, 338.04]
KEYNOTE-010,2016b	15	343	0	309	49.8%	29.21 [1.74, 490.22]
**Total (95% CI)**		**682**		**618**	**100.0%**	**24.44 [3.31, 180.61]**
Total events	25		0			

Heterogeneity: Chi² = 0.04, df = 1 (P = 0.85); I² = 0%
Test for overall effect: Z = 3.13 (P = 0.002)

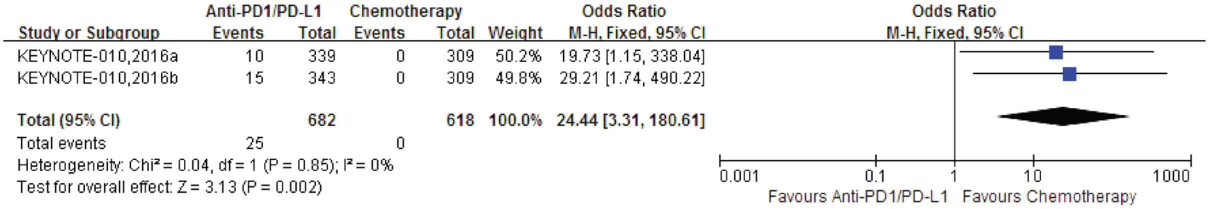

Supplementary Figure 10: The forest plot of hyperthyroidism in advanced NSCLC patients who received anti-PD1/PD-L1 antibody therapy compared to docetaxel.

Study or Subgroup	Anti-PD1/PD-L1 Events	Anti-PD1/PD-L1 Total	Chemotherapy Events	Chemotherapy Total	Weight	Odds Ratio M-H, Fixed, 95% CI
KEYNOTE-010,2016a	25	339	1	309	49.7%	24.52 [3.30, 182.10]
KEYNOTE-010,2016b	23	343	1	309	50.3%	22.14 [2.97, 164.93]
**Total (95% CI)**		**682**		**618**	**100.0%**	**23.32 [5.64, 96.37]**
Total events	48		2			

Heterogeneity: Chi² = 0.00, df = 1 (P = 0.94); I² = 0%
Test for overall effect: Z = 4.35 (P < 0.0001)

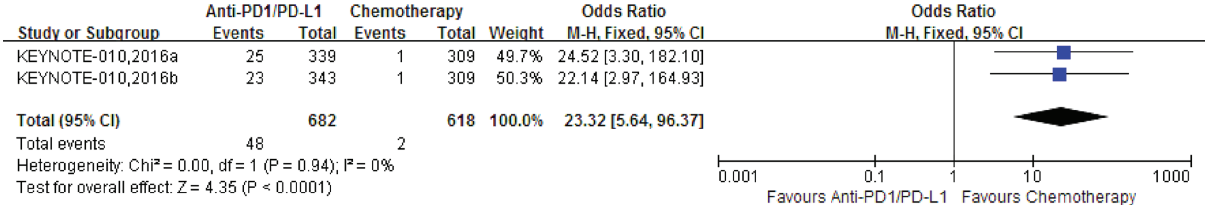

Supplementary Figure 11: The forest plot of hypothyroidism in advanced NSCLC patients who received anti-PD1/PD-L1 antibody therapy compared to docetaxel.
